# Recent progress in unraveling the molecular mechanisms of anthocyanin biosynthesis and regulation in tomato

**DOI:** 10.1093/hr/uhag057

**Published:** 2026-02-23

**Authors:** Yuanyuan Kong, Aiyin Cui, Xuemei Hou, Yali Zhu, Weibiao Liao

**Affiliations:** College of Horticulture, Gansu Agricultural University, 1 Yinmen Village, Anning District Lanzhou 730070, China; College of Horticulture, Gansu Agricultural University, 1 Yinmen Village, Anning District Lanzhou 730070, China; College of Horticulture, Gansu Agricultural University, 1 Yinmen Village, Anning District Lanzhou 730070, China; College of Horticulture, Gansu Agricultural University, 1 Yinmen Village, Anning District Lanzhou 730070, China; College of Horticulture, Gansu Agricultural University, 1 Yinmen Village, Anning District Lanzhou 730070, China

## Abstract

Tomato (*Solanum lycopersicum*) is one of the most economically important vegetable crops worldwide. Fruit quality is a critical determinant of consumer preference and market value, with color being the primary visual trait. While carotenoids impart red pigmentation, anthocyanins enable the accumulation of deep purple and blue hues. Although anthocyanins have been widely studied, a comprehensive understanding of their biosynthesis and regulation in tomato is still lacking. This review therefore synthesizes current knowledge to outline the molecular mechanisms underlying these processes. We highlight the central role of the MYB–bHLH–WD40 transcriptional activation complex. Additionally, we discuss the multilayered regulatory network involving other transcription factors, such as the bZIP family members SlHY5 and SlAREB1, BBX proteins, and others. Furthermore, we elaborate on post-transcriptional and post-translational regulatory mechanisms, which fine-tune anthocyanin accumulation. Finally, we outline current challenges and future directions for enhancing tomato anthocyanins. This review serves the dual purpose of providing an updated theoretical foundation for genetic improvement in tomato and offering a regulatory framework applicable to other horticultural crops.

## Introduction

Anthocyanins, the largest and most important group of water-soluble pigments in the flavonoid family, are widely accumulated in plant tissues such as fruits, flowers, and leaves [1, 2]. Beyond their vibrant colors, which attract pollinators and seed dispersers, anthocyanins play a critical role in plant adaptation to environmental stresses [3, 4]. As potent antioxidants, they scavenge reactive oxygen species (ROS) generated under abiotic stresses such as high light and low temperature, thereby mitigating oxidative damage in plants [5–8]. Beyond direct ROS scavenging, anthocyanins can mitigate heavy metal toxicity through metal chelation, forming complexes with ions such as chromium (Cr) and arsenic (As) and facilitating their sequestration into vacuoles [9, 10]. Notably, this protective accumulation is often regulated by certain signaling molecules. Under Cr stress, melatonin upregulates key gene expression to promote anthocyanin biosynthesis [11]; whereas under As stress, combined melatonin and hydrogen sulfide treatment synergistically enhance the accumulation of anthocyanins and polyphenols, and activate related biosynthetic genes [12]. These findings collectively indicate that anthocyanin-mediated alleviation of heavy metal stress relies not only on its antioxidant activity and direct chelation ability but is also integrated into broader plant signaling regulatory networks. Recently, anthocyanins have gained significant attention for their nutraceutical properties [13]. Dietary intake of anthocyanins has been linked to anti-inflammatory, anticancer, anti-obesity activities, as well as the prevention of cardiovascular diseases and diabetes, making them highly valued phytonutrients [2, 14]. Consequently, enhancing anthocyanin content in common vegetables and fruits to improve their nutritional and health benefits has become a major research focus, offering consumers healthier dietary options.

Tomato (*Solanum lycopersicum*) is a globally important horticultural crop widely cultivated and consumed. Recent breeding efforts have successfully developed tomato varieties capable of synthesizing and accumulating high levels of anthocyanins in their fruits [15–17]. These strategies often rely on novel alleles introduced from wild tomato relatives. Among the most widely used genetic factors are the *Anthocyanin fruit* (*Aft*) and *atroviolacea* (*atv*) loci [18]. The *Aft* trait, derived from *S. chilense*, enables anthocyanin production in the tomato fruit peel under strong light or low temperatures, resulting in purple spots on the fruit [19]. Most cultivated tomato varieties lack significant accumulation of anthocyanins in fruits due to functional mutations in key regulatory genes. But certain wild tomato relatives, such as *S. peruvianum* and purple lines like Indigo Rose (InR), possess the capacity to synthesize anthocyanins in fruits, hypocotyls, or leaves [20, 21]. This core difference arises from genetic variations in the anthocyanin synthesis and regulatory pathways. During development, anthocyanin accumulation in tomato is often associated with fruit ripening or stress responses, a process tightly regulated by key enzyme genes and transcription factors (TFs) in response to environmental signals [12, 22].

In recent years, advancements in molecular biology techniques have significantly broadened the scope anthocyanin research. The focus has shifted from model plants such as Arabidopsis to economically important crops like tomato. This shift has enabled a deeper exploration of the molecular mechanisms governing anthocyanin biosynthetic pathways and their regulatory networks. This review systematically synthesizes the latest progress in tomato anthocyanin research, centering on three core areas: (i) the anthocyanin biosynthetic pathway, (ii) the transcriptional regulatory network involving MBW complex and other TFs families, and (iii) post-transcriptional and post-translational regulatory mechanisms, including alternative splicing, miRNA and ubiquitination modifications. By addressing these aspects, this review aims to provide theoretical insights into the intricate of plant secondary metabolism and to offer practical guidance for the genetic enhancement of high-anthocyanin tomato varieties.

## The anthocyanin biosynthetic pathway and the structural genes

**Figure 1:**
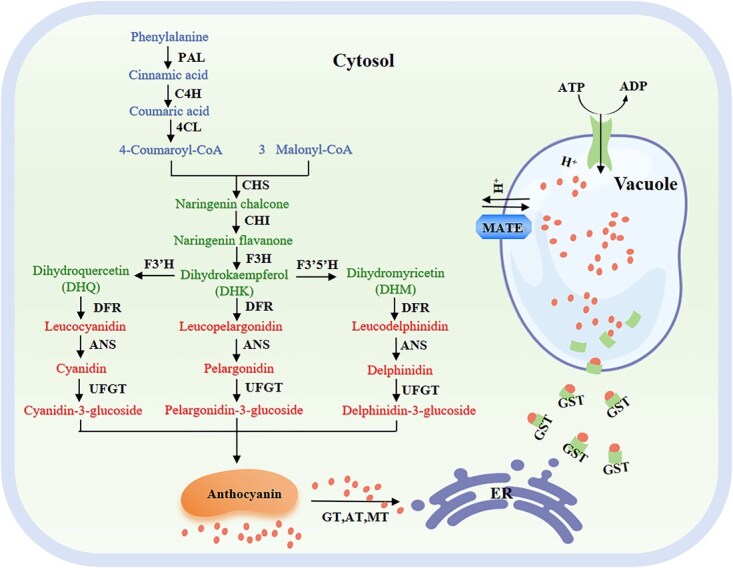
Schematic diagram of the anthocyanin biosynthesis pathway in plants. The pathway is divided into three primary stages: (1) the early stage of anthocyanin synthesis, including the formation of precursors such as phenylalanine and the conversion to chalcones. (2) the intermediate stage, where chalcones are transformed into flavanones and further modified into dihydroflavonols. (3) the final stage, where dihydroflavonols are converted into anthocyanidins, the core structures of anthocyanins. Additionally, the figure illustrates the vacuolar transport and storage of anthocyanins, a critical step ensuring their accumulation in plant tissues. Abbreviations: PAL, phenylalanine ammonia-lyase; C4H, cinnamate 4-hydroxylase; 4CL, 4-coumarate: CoA ligase; CHS, chalcone synthase; CHI, chalcone isomerase; F3H, flavanone 3-hydroxylase; F3′H, flavonoid 3′-hydroxylase; F3′5′H, flavonoid 3′, 5′-hydroxylase; DFR, dihydroflavonol 4-reductase; ANS, anthocyanidin synthetase; UFGT, uridine diphosphate-glucose: flavonoid-3-O-glucosyltransferase; ER, endoplasmic reticulum; GST, Glutathione S-transferases.

Anthocyanin biosynthesis in plants has been intensively investigated, and its pathways are well characterized. This process represents a branch of the broader flavonoid pathway, which originates from the highly conserved phenylpropane pathway [23, 24]. The entire biosynthetic process can be divided into three stages (Fig. 1).

The first stage involves the conserved phenylpropanoid pathway, where the starting substrate phenylalanine is converted to the key co-precursor 4-coumaroyl-CoA through a sequential catalytic reaction mediated by phenylalanine ammonia-lyase (PAL), cinnamate 4-hydroxylase (C4H), and 4-coumarate CoA ligase (4CL). The second stage is the formation of the flavonoid core skeleton. Here, chalcone synthase (CHS) catalyzes the condensation and cyclisation of 1 molecule of 4-coumaroyl coenzyme A with 3 molecules of malonyl coenzyme A to form naringenin chalcone. This intermediate is then isomerized to naringenin (flavanone) by chalcone isomerase (CHI). Naringenin is subsequently hydroxylated by flavanone 3-hydroxylase (F3H) to produce dihydrokaempferol (DHK). Flavonoid 3′-hydroxylase (F3′H) and flavonoid 3′,5′-hydroxylase (F3′5′H) further act on DHK or its derivatives to introduce hydroxyl groups in the B-ring, yielding dihydroquercetin (DHQ), a cyanidin precursor, and dihydromyricetin (DHM), a precursor of delphinidin. The genes encoding these enzymes (CHS, CHI, F3H, F3′H, F3′5′H) are collectively referred to as early biosynthesis genes (*EBGs*), which provide precursors for a variety of flavonoids, including anthocyanins. The third stage is the specific synthesis of anthocyanin glycosides. In this phase, dihydroflavonol 4-reductase (DFR) reduces dihydroflavonols (DHK, DHQ, DHM) to their corresponding leucoanthocyanidins. These colorless intermediates are then oxidatively dehydrogenated by anthocyanidin synthase (ANS) or leucoanthocyanidin dioxygenase (LDOX) to generate unstable anthocyanidins (delphinidin, pelargonidin, and cyanidin). The key enzyme UDP-glucoside: flavonoid-3-O-glucosyltransferase (UFGT) subsequently glycosylates these anthocyanidins, primarily at the C3 position, to form stable anthocyanin glycosides such as delphinidin-3-glucoside, pelargonidin-3-glucoside, and cyanidin-3-glucoside. Further structural diversity is achieved through modifications by O-methyltransferases (OMTs) and acyltransferases (AT), which mediate processes like methylation and acylation. The genes encoding DFR, ANS/LDOX, and UFGT are called late biosynthetic genes (*LBGs*). The enzymes acting downstream of LBGs, which modify the anthocyanin structure, are termed modification enzymes. These genes expression is typically highly and positively correlated with the accumulation of anthocyanins, directly determining the synthesis of these pigments [25, 26]. Notably, the substrate specificity of key enzymes influences the final product composition. For example, the preference of DFR for the utilization of DHM in tomato leads to a predominance of delphinidin-type anthocyanin glycosides in their fruit [27].

After being synthesized on the endoplasmic reticulum (ER) surface of the cytoplasm, anthocyanin glycosides undergo a series of modifications, including glycosylation, acylation, methylation and other modifications, to improve their stability (Fig. 1). Subsequently, these modified anthocyanins are translocated and stored in the acidic environment of vacuoles, where they achieve stable color development. This process is facilitated by various mechanisms, such as vacuole-mediated transport, glutathione S-transferase (GST)-mediated transport, or active transport by flavonoids/H^+^ antitranslocator proteins [24, 28, 29]. Defects in this process can result in phenotypic abnormalities. For instance, a mutation in the *SlGSTAA* gene in tomatoes causes anthocyanin deficiency in seedlings, resulting in a green hypocotyl phenotype instead of the typical anthocyanin-pigmented appearance [30]. In addition, structural genes such as *SlCHI*, *SlF3H*, and *SlDFR* have been identified in tomatoes through fine mapping or map-based cloning approaches [31–33]. These genes play critical roles in the anthocyanin biosynthesis pathway, further elucidating the genetic basis of anthocyanin production in tomato plants. Elevated expression of key structural genes (e.g. *F3*′*5*′*H*, *DFR*, *ANS*) in tomato fruit mutants drives the increased accumulation of anthocyanin glycoside pigments [34]. Research has demonstrated that differential expression of these structural genes is often the most direct factor influencing variations in anthocyanin content. For example, knockdown of *CHS* transcripts in tomato through RNA interference reduces the expression of *Chs1* and *Chs2* transcripts, as well as CHS enzyme activity, resulting in anthocyanin deficiency and a cyan-colored peel phenotype in tomato fruits [35]. As research advances, the roles of these key structural genes in anthocyanin biosynthesis are becoming increasingly clarified, providing a strong theoretical foundation for the genetic improvement of fruit coloration.

## Effects of TFs on anthocyanin biosynthesis

**Figure 2:**
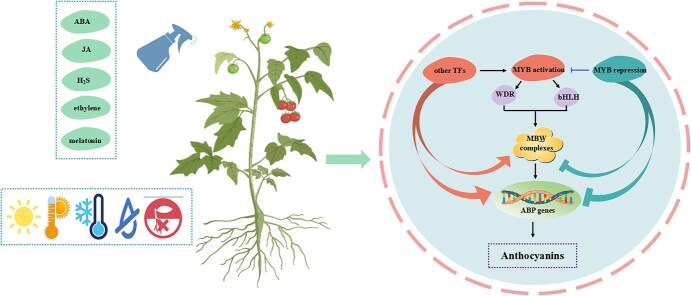
Schematic diagram of anthocyanin biosynthesis regulation in tomatoes. Exogenous signaling molecules, such as abscisic acid (ABA), jasmonic acid (JA), hydrogen sulfide (H₂S), ethylene, and melatonin, alongside environmental factors including light, temperature extremes (high and low), drought, and heavy metal stress, collectively trigger the anthocyanin regulatory pathway. These diverse signals modulate the pathway by either activating or suppressing a cascade of key TFs, which in turn regulate the expression of anthocyanin biosynthetic pathway (ABP) genes. This intricate regulatory network ultimately governs the synthesis and accumulation of anthocyanins, ensuring their production in response to both internal and external stimuli.

While the structural genes encoding the biosynthetic enzymes are essential, their expression alone is insufficient to determine the final anthocyanin accumulation. The spatiotemporal specificity and accumulation intensity of this pathway are primarily governed by a complex transcriptional regulatory network. Within this network, the MYB-bHLH-WD40 (MBW) transcription complex occupies a central position. It directly orchestrates the entire biosynthetic pathway by activating downstream structural genes (Fig. 2). This complex comprises R2R3-MYB TFs, bHLH TFs, and WD40 repeat proteins [21]. In tomato, several R2R3-MYBs, such as SlAN2, SlAN2-like, and SlANT1, have been identified as critical regulators of anthocyanin accumulation [18, 36, 37]. bHLHs also play a role in anthocyanin biosynthesis by directly promoting structural genes’ expression and enhancing anthocyanin accumulation through interactions with MYBs [38]. WD40 proteins, characterized by 4–10 random WD repeat domains, lack catalytic activity but serve as platforms for assembling macromolecular protein complexes and facilitating their transport to the nucleus for further function [39, 40]. Notably, the WD40 protein SlAN11 plays a crucial role within this complex by directly interacting with the bHLH component, though it does not bind the MYB protein [39]. Furthermore, overexpression of *SlAN11* significantly enhances anthocyanin accumulation in leaves and stems, whereas RNAi lines suppressing its expression exhibit nearly no anthocyanin accumulation in these tissues [39]. These results collectively demonstrate that SlAN11 functions in coordination with bHLH and MYB proteins to positively regulate anthocyanin biosynthesis in tomato.

## MYB

Defined as the largest family of plant TFs, MYB TFs are widely present across species and govern a broad spectrum of physiological processes spanning growth, development, secondary metabolism, and stress resistance. MYB TFs dominate regulatory MBW complexes due to their highly conserved DNA-binding domain (R domain, consisting of 51–52 amino acids) [20, 41]. Based on the number of R domains, MYB TFs are classified into R3-MYB, R2R3-MYB, and R1R2R3-MYB subfamilies [42]. A cluster of R2R3-MYB genes located at the Aft locus on chromosome 10, including *SlANT1*, *SlANT1-*like (*SlMYB28*), *SlAN2-*like, and *SlAN2* (*SlMYB75*), are key positive regulators of anthocyanin accumulation. Overexpression of these genes enhances anthocyanin accumulation [13, 37, 43–47]. CRISPR/Cas9-mediated knockout experiments confirmed that *SlAN2-*like is a central determinant of anthocyanin accumulation in fruits [18]. The expression of *SlAN2* is strongly induced by external environmental signals, positioning it as the primary MYB protein mediating environmentally induced anthocyanin synthesis in tomato. *SlAN2* activates the transcription of bHLH factors *SlAN1* and *SlJAF13*, which in turn upregulate anthocyanin structural genes such as *SlDFR*. Overexpression of *SlAN2* not only upregulates structural genes in the anthocyanin biosynthetic pathway and promotes anthocyanin accumulation but also enhances stress resistance in transgenic tomato plants. Compared to wild-type, *SlAN2* overexpressing plants alleviate photo-inhibition and oxidative damage under 40°C heat stress, significantly improving thermotolerance [48]. Similarly, *SlAN2* plays a critical role in low-temperature stress response. Overexpression of *SlAN2* leads to increased anthocyanin accumulation and enhanced chilling tolerance at 4°C, whereas its silencing inhibits anthocyanin biosynthesis [18, 22]. Further research reveals that while *SlAN2* and *SlANT1* are functionally redundant, their expression patterns and physiological roles differ significantly. In contrast, *SlANT1* exhibits very low expression and no inductive response under various stresses, and its silencing does not affect anthocyanin accumulation, indicating a minor role in this regulatory process [37]. It is noteworthy that the regulatory function of MYB TFs in anthocyanin biosynthesis is conserved and significant in wild tomato relatives. In *S. peruvianum*, the expression of MYB TFs *SpAN2* (a homolog of cultivated tomato *SlAN2*) is strongly induced by drought stress and UV-B radiation [49]. Drought stress induces both the accumulation of *SpAN2* transcripts and anthocyanins in tomato fruits. This coordinated upregulation suggests a direct regulatory function for *SpAN2* in promoting anthocyanin synthesis under water-deficient conditions. Furthermore, *SpAN2* transcript levels peak during periods of strongest daily UV-B radiation and are further upregulated under controlled UV-B treatment, confirming that UV-B as a key environmental signal regulating *SpAN2* expression. *SpAN2* promotes anthocyanin accumulation under both drought and UV-B stresses by directly or indirectly activating the downstream anthocyanin biosynthetic gene *SpDFR* [49].

Anthocyanin biosynthesis in tomato is subject to negative feedback regulation (Fig. 2), involving three R3-MYBs (*SlMYBATV*, *SlMYBATV-*like, *SlTRY*) and four R2R3-MYBs (*SlMYB3*, *SlMYB7*, *SlMYB32* (*SlTHM27*), *SlMYB76*) [13, 44, 50–52]. The repressive function of SlMYBATV was confirmed through the CRISPR/Cas9 approach. It inhibits anthocyanin synthesis by competing with *SlAN2-like* for binding to *SlAN1* and *SlJAF13*, disrupting the MBW complex assembly [18, 21, 53]. Overexpression of *SlTRY* reduces anthocyanin accumulation [52, 54], whereas silencing this gene increases anthocyanins content [55]. Additionally, transient overexpression of *SlMYB7* can inhibit anthocyanin accumulation and downregulate the expression of multiple biosynthetic genes. On one hand, SlMYB7 directly binds to the promoters of structural genes, actively repressing their transcription. On the other hand, it interacts with the bHLH proteins SlJAF13 and SlAN1, which participate in the formation of the MBW complex, thereby blocking the assembly of the complex and passively interfering with anthocyanin synthesis [56]. This dual action, targeting both the endpoint (gene promoters) and the upstream activator (the MBW complex itself), suggests that SlMYB7 may function in a negative feedback loop to coregulate and fine-tune anthocyanin biosynthesis, providing a robust homeostatic control mechanism. Notably, SlTHM27 acts as a negative regulator of anthocyanin accumulation and cold response. It directly represses the transcription of *SlGAD2*, which encodes glutamate decarboxylase, a key enzyme in gamma-aminobutyric acid (GABA) biosynthesis. Under cold stress, *SlTHM27* expression is suppressed, lifting its inhibition on *SlGAD2* and leading to GABA accumulation. GABA enhances antioxidant enzyme activity, alleviates oxidative damage, and promotes the expression of key anthocyanin biosynthetic genes (*SlCHS*, *SlF3′H*, *SlDFR*), ultimately improving chilling tolerance. The *slthm27* mutant exhibits higher *SlGAD2* expression, GABA and anthocyanin content, antioxidant capacity, and cold resistance, confirming *SlTHM27*’s role in negatively regulating cold stress response through the SlGAD2-mediated GABA and anthocyanin pathways [57].

Beyond well-studied MYBs, the functions of other family members in tomato anthocyanin biosynthesis are less clear. For instance, sequence analysis indicates that SlMYB7-like harbors the conserved bHLH-interaction motif within the MBW complex [58]. It has been speculated that SlMYB7-like may interact with *SlGL3* to activate the anthocyanin pathway in tomato [59]. Additionally, SlMYB48-like has been proposed to participate indirectly, possibly by modulating the stability of the MBW complex [59]. Similarly, SlMYB12, a functional homolog of *AtMYB12*, upregulates early flavanol biosynthetic genes, *PAL*, *CHS*, *CHI*, *F3H*, and *FLS*, but does not activate the anthocyanin-specific gene *DFR*. Silencing of *SlMYB12* results in pink tomato fruits with reduced accumulation of the yellow flavonoid naringenin chalcone [60, 61]. The precise regulatory contributions and mechanisms of these MYB factors in anthocyanin biosynthesis await further investigation.

Additionally, anthocyanin biosynthesis is negatively regulated by ethylene. In the purple tomato cultivar InR, ethylene treatment downregulates key biosynthetic genes (*SlF3′5′H*, *SlDFR*, *SlANS*) and core positive regulators (*SlAN2-*like, *SlAN1*) [36]. Transcriptome analysis further identifies ERF, MYB, and bHLH family TFs whose expression correlates with anthocyanin content, suggesting their potential role in mediating ethylene-induced suppression of anthocyanin synthesis [36]. These findings highlight the ethylene signaling pathway as a negative regulator of anthocyanin biosynthesis during fruit ripening.

## Basic helix–loop–helix

The basic helix–loop–helix (bHLH) family is the second largest group of TFs in plants. These proteins typically contain two conserved domains, a basic region that binds to E-box and G-box cis-elements, and a helix–loop–helix (HLH) domain responsible for dimerization [62]. In tomato, the bHLH factors *SlJAF13* and *SlAN1* (*SlTT8*) are essential components of the MBW transcriptional activation complex and act as positive regulators of anthocyanin biosynthesis (Fig. 2). *SlJAF13* serves as an upstream key regulator, directly binding to the G-box motif in the *SlAN1* promoter to activate its transcription. It also forms a functional MBW complex with the *SlAN2-*like and the *SlAN11*, initiating the expression of anthocyanin structural genes [20, 38, 63, 64]. Similarly, SlAN1 forms a functional MBW complex with *SlAN2* and *SlAN11* to directly activate the transcription of anthocyanin structural genes, strongly promoting anthocyanin accumulation in both vegetative tissues and fruits. Notably, *SlAN1* function is highly dependent on light intensity. Its expression and anthocyanin synthesis are significantly induced under strong light but fail to initiate effectively under low light conditions [65]. Furthermore, *SlAN1* interacts with *SlMYBL2*, a negative feedback regulator that competitively binds to the bHLH component, inhibiting the MBW complex assembly and preventing excessive anthocyanin accumulation [65] .

The JA signaling pathway suppressor *SlJAZ2* directly interacts with *SlJAF13*, *SlAN1*, and *SlAN11*, interfering with the assembly of the MBW complex and suppressing its transcriptional activity. Moreover, *SlJAF13* interacts with the core JA signaling TF *SlMYC2* to form a heterodimer, which subsequently inhibits *SlMYC2*’s ability to activate *SlJAZ2* transcription. This interaction establishes a precise negative feedback loop that prevents over-accumulation of SlJAZ2 and fine-tunes anthocyanin biosynthesis [38]. Furthermore, *SlGL3*, a homolog of the Arabidopsis bHLH TF *AtGL3*, has been reported to negatively regulate anthocyanin accumulation [54, 66]. Under low-phosphate (Pi) stress, *SlGL3* expression is significantly reduced in tomato seedlings, concomitant with a marked increase in anthocyanin content [66]. This suggests that *SlGL3* may participate in the negative regulation of anthocyanin biosynthesis through a repressive cascade.

## Other TFs

In addition to the core MYB, bHLH, and WD40 proteins, other TF families such as bZIP, B-box (BBX) also play pivotal roles in regulating the anthocyanin metabolic pathway (Fig. 2). These TFs interact with the MBW complex, modulating its activity either upstream or downstream, thereby contributing to the intricate architecture of the anthocyanin regulatory network. The bZIP factor *SlHY5*, a central component of the light signaling pathway, activates transcription by directly binding to G-box/ACE elements in the promoters of anthocyanin structural genes (*SlCHS*, *SlDFR*) and the regulatory gene *SlAN2-*like [67–71]. Upon light induction, SlHY5 promotes anthocyanin accumulation by directly activating the master regulator *SlAN2*-like. This leads to the upregulation of downstream biosynthetic genes and *SlAN1*; simultaneously, it induces the repressor *SlMYBATV*, forming a regulatory network [21]. Furthermore, experimental evidence has demonstrated that mutation of *SlHY5* leads to severe impairment of anthocyanin synthesis in tomato fruits even under optimal temperature conditions, confirming the indispensable role of SlHY5 in integrating light and temperature signals to regulate anthocyanin accumulation in tomato fruits [68]. Another bZIP factor, *SlAREB1*, has been shown to positively regulate anthocyanin synthesis in response to low-temperature stress through an ABA-dependent pathway. Xu et al. have demonstrated that *SlAREB1* directly binds to ABRE *cis*-elements in the promoters of *SlDFR* and *SlF3′5′H*, activating their transcription and significantly promoting anthocyanin accumulation under cold conditions [72]. Meanwhile, overexpression of *SlAREB1* results in higher anthocyanin content in tomatoes under low temperature, whereas silencing this gene markedly impairs anthocyanin synthesis.

Beyond the bZIP family, SlBBX20 directly activates *SlDFR* expression to promote anthocyanin biosynthesis, and its overexpression leads to increased anthocyanin accumulation [73]. In contrast, *SlBBX24*, which interacts with both *SlAN2-*like and *SlAN2*, functions as a negative regulator. Silencing *SlBBX24* leads to the downregulation of structural genes (*SlCHS1*, *SlDFR*, *SlANS*) and positive regulators (*SlAN1*, *SlAN2-*like, *SlAN11*), while upregulating negative regulators (*SlMYBATV*, *SlTRY*, *SlMYB76*) [71]. Additionally, SlCOL1 (*SlBBX3*) directly activates the *SlAN1* promoter and interacts with *SlAN1* under short-day (SD) and low-temperature conditions. This interaction forms a regulatory complex that synergistically enhances the expression of anthocyanin biosynthetic genes, significantly increasing anthocyanin accumulation and ROS scavenging capacity, thereby aiding the plant’s response to cold stress [74]. Anthocyanin accumulation was significantly reduced in *SlCOL1* CRISPR/Cas9 mutants relative to wild-type plants, especially under SD at suboptimal low-temperature conditions [74]. Furthermore, *SlWRKY44* may regulate anthocyanin synthesis through interaction with *SlAN11* [71]. Overexpression of *SlCSN5-2* significantly suppresses anthocyanin accumulation, while its silencing results in hyper-accumulation in calli at the cost of normal growth, demonstrating its essential role in development [73].

A recent study has identified *SlPHL1* as a key positive regulator of anthocyanin biosynthesis under low-phosphate (LP) stress [75]. *SlPHL1* directly binds to P1BS *cis*-elements in the promoters of critical anthocyanin biosynthetic genes *SlF3H* and *SlLDOX*, activating their transcription. Overexpression of *SlPHL1* significantly increases anthocyanin content under LP conditions, whereas its silencing attenuates anthocyanin synthesis and the expression of related genes [75]. These findings highlight the crucial role of *SlPHL1* in phosphate starvation-induced anthocyanin synthesis in tomato and suggest that other PHR family members may co-regulate this network. Transcriptome-related network analyses have identified additional TFs potentially involved in anthocyanin biosynthesis [55, 71], indicating that the understanding of the gene regulatory network governing tomato anthocyanin metabolism remains incomplete and requires further exploration.

## Post-transcriptional and post-translational regulation

### Alternative splicing and aberrant splicing

Alternative splicing (AS) is a key post-transcriptional regulatory mechanism in plants that modulates gene expression and ultimately influences plant morphology and function [76]. Aberrant splicing refers to incorrect pre-mRNA processing caused by genetic mutations (e.g. base substitutions, insertions), which typically produces nonfunctional or truncated proteins rather than functionally differentiated isoforms [77]. In tomato, both regulated AS and sequence variation-mediated aberrant splicing have been reported to influence anthocyanin biosynthesis.

**Figure 3:**
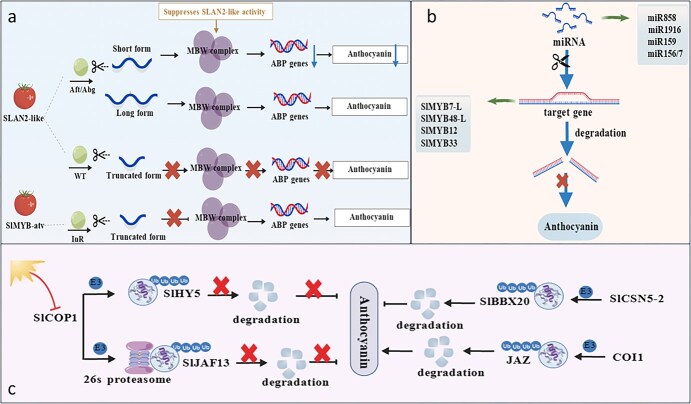
Post-transcriptional (a and b) and post-translational (c) regulation of anthocyanin biosynthesis in tomato. (a) the TF SlAN2-like generates multiple protein isoforms through alternative splicing and aberrant splicing. These isoforms regulate the expression of Anthocyanin Biosynthesis Pathway (ABP) genes, thereby influencing anthocyanin accumulation. SlMYB-atv is truncated and no longer able to repress the action of the MBW complex. (b) miR858 and miR1916 suppress anthocyanin accumulation by targeting and inhibiting the expression of key TFs, including SlMYB48-like, SlMYB7-like, and SlMYB12. miR156/7 and miR159 may also inhibit anthocyanin accumulation by repressing the expression of *ABP* genes. (c) SlCOP1, COI1, and SlCSN5-2 promote the degradation of specific proteins, including SlHY5, SlJAF13, SlBBX20, and SlJAZ proteins, thereby affecting anthocyanin accumulation. Abbreviations: Constitutive Photomorphogenic 1 (COP1), CORONATINE INSENSITIVE1 (COI1), COP9 signalosome (CSN), Elongated hypocotyl 5 (HY5), JA ZIM-domain (JAZ), B-box 20 (BBX20).

In most cultivated tomatoes, a G-to-A mutation at the 5′ splice site of the second intron in the *SlAN2-*like allele leads to aberrant pre-mRNA splicing (Fig. 3). This aberrant splicing causes a frameshift mutation, introducing a premature stop codon and producing a truncated protein lacking the R3 domain, which is essential for interaction with bHLH. Consequently, the MBW complex cannot form, and anthocyanin biosynthesis is disrupted [20, 21, 44]. Additionally, *SlMYBATV* carries an insertion mutation in purple-fruited lines such as *InR*. This mutation introduces a premature stop codon, resulting in a truncated nonfunctional protein that is unable to compete with SlAN2-like for SlAN1 binding. The loss of function in *SlMYBATV*, combined with the presence of a functional *SlAN2-*like allele, enables potent anthocyanin accumulation in *InR* fruits [21].

In contrast, wild tomato species such as *S. chilense* and introgression lines like *Aft* and *Abg* carry alleles with an intact splice site. This enable correct splicing and the production of a full-length, functional SlAN2-like protein, which is capable of activating anthocyanin synthesis [21]. Notably, in the *Abg* line, *SlAN2-*like undergoes AS to produce two transcript isoforms: a long, fully functional form and a shorter variant with a 27-amino acid deletion near the S6B motif. Although both isoforms localize to the nucleus and retain transcriptional activity, the shorter protein shows significantly reduced activation capacity [44]. The differential expression of these long and short transcripts, coupled with the distinct activities of the two protein isoforms, is hypothesized to enable dynamic regulation of anthocyanin production in response to developmental or environmental cues. However, further experimentation is required to fully elucidate this process. In summary, the discovery of AS in tomato significantly expands our understanding of anthocyanin regulation, highlighting the role of transcript diversity in the fine-tuning of metabolic responses.

## MicroRNA

MicroRNAs (miRNAs) are a class of endogenous small noncoding RNAs, approximately 20–24 nucleotides in length, that negatively regulate protein-coding gene expression by either cleaving target mRNAs or inhibiting their translation [78, 79]. Numerous studies have demonstrated that plant miRNAs play pivotal regulatory roles in various biological processes, including development, primary and secondary metabolism, and stress responses [80, 81]. In tomato, several miRNAs have been identified as key regulators of anthocyanin biosynthesis, modulating anthocyanin accumulation by targeting both TFs and structural genes involved in the pathway (Fig. 3). Among these, miR858 is a well-characterized negative regulator of anthocyanin biosynthesis in tomato. By directly targeting and cleaving the transcripts of *SlMYB7-like* and *SlMYB48-*like [59], it establishes a direct post-transcriptional regulatory link. To further validate the function of miR858, Jia et al. employed a short tandem target mimic STTM858 to specifically silence endogenous miR858, generating transgenic tomato plants overexpressing *STTM858*. In these plants, endogenous miR858 levels are significantly reduced, accompanied by a marked increase in *SlMYB7-*like transcripts [59]. Concurrently, the expression of key structural genes in the anthocyanin biosynthetic pathway is substantially upregulated. Notably, the expression of *SlMYB48-like* is also induced, and 5′ RACE experiments confirmed for the first time that its transcript is directly cleaved by miR858 [59]. Although the precise mechanism by which *SlMYB48-*like regulates anthocyanin synthesis remains to be fully elucidated, it is hypothesized to act indirectly by modulating upstream genes in the phenylpropanoid pathway or by affecting the stability of the MBW complex. Importantly, STTM858-mediated silencing of miR858 does not induce the expression of *SlMYB12* [59], indicating that the regulation of anthocyanin biosynthesis by miR858 is specific to *SlMYB7-*like and *SlMYB48-*like, rather than a general activation of all MYB TFs involved in flavonoid metabolism. In addition to miR858, sly-miR1916 has been identified as another negative regulator of anthocyanin biosynthesis in tomato [82]. Transgenic tomato plants overexpressing sly-miR1916 shows reduced expression of predicted target genes, including *MYB12*. Conversely, transgenic lines with silenced sly-miR1916 exhibit significantly increased *MYB12* expression and higher anthocyanin levels compared to wild-type controls. 5′ RACE experiments further validate the direct regulatory interaction between sly-miR1916 and its target, identifying a cleavage site in the *MYB12* mRNA downstream of the predicted sly-miR1916 binding site [82]. Collectively, these findings suggest that sly-miR1916 plays a crucial role in regulating anthocyanin biosynthesis, potentially through direct or indirect modulation of *MYB12* expression.

Other miRNAs are also directly or indirectly involved in the regulation of anthocyanin metabolism in tomato. Overexpression of *MIR156/7* in tomato leads to increased accumulation of naringenin chalcone [83]. This finding suggests that miR156/7 acts at specific nodes of the flavonoid pathway to modulate metabolic flux, potentially directing it toward anthocyanin synthesis during fruit development, although its direct targets and precise mechanism of action in this context require further investigation. Additionally, the expression of sly-miR159 in tomato decreases under drought stress, and its target gene, *SlMYB33*, induces the expression of *SlP5CS*, a key enzyme gene in proline biosynthesis, thereby promoting the accumulation of osmoprotectants [84]. Although a direct link between sly-miR159 and anthocyanin synthesis has not been firmly established, this observation highlights the potential role of miRNAs in integrating stress responses with secondary metabolism.

## Ubiquitination

Anthocyanin biosynthesis in tomato is tightly regulated at the post-translational level, with ubiquitin-mediated protein degradation serving as a key regulatory mechanism that directly affects the stability of critical transcriptional regulators and signaling components. Constitutive Photomorphogenic 1 (SlCOP1), a canonical RING-type E3 ubiquitin ligase [85–87], acts as a central negative regulator of anthocyanin biosynthesis by targeting multiple positive regulators for degradation (Fig. 3). Liu et al. have shown that upon activation by blue light, the cryptochrome photoreceptor CRY1a inhibits SlCOP1 activity, thereby preventing the degradation of SlHY5 [88]. This leads to the accumulation of SlHY5, which activates the transcription of downstream anthocyanin biosynthetic genes *CHS1*, *CHS2*, and *DFR*. Notably, the regulatory function of SlCOP1 is further integrated with the COP1-SPA-DET1 module [89, 90]. De-etiolated 1 (DET1) is a core component of the CDD complex and is essential for the assembly of the functional COP1-SPA E3 ubiquitin ligase complex [91–94], which serves as another key regulator in anthocyanin biosynthesis. Under normal temperature conditions (23°C), SlCOP1 is predominantly localized in the cytoplasm and cannot interact with nuclear SlHY5. In contrast, under high temperature (30°C), SlCOP1 translocates into the nucleus, where it associates with SPA proteins and the CDD complex to form an active E3 ubiquitin ligase complex [68]. This complex specifically targets SlHY5 for ubiquitination and subsequent proteasomal degradation. The resulting sharp decline in SlHY5 protein levels prevents the activation of anthocyanin biosynthetic genes, ultimately suppressing anthocyanin accumulation [68]. Importantly, the *hp2* mutant, which carries a loss-of-function mutation in *DET1*, disrupts the function of this E3 ligase complex. Consequently, SlHY5 remains stable even under high temperature, enabling sustained anthocyanin accumulation and confirming the critical role of DET1 in temperature sensing and anthocyanin regulation in tomato [68]. Furthermore, a recent study revealed that SlCOP1 directly interacts with the SlJAF13, promoting its polyubiquitination and degradation via the 26S proteasome pathway [95]. Overexpression of *SlCOP1* significantly reduces SlJAF13 protein levels and decreases anthocyanin accumulation. However, treatment with a proteasome inhibitor stabilizes SlJAF13 and prevents its degradation even in the presence of SlCOP1, confirming that SlCOP1-mediated SlJAF13 degradation is proteasome-dependent [95]. Notably, the stability of the SlJAF13 protein is light-regulated and light inhibits SlCOP1-mediated degradation, thereby stabilizing SlJAF13, while its mRNA levels remain unaffected by light, indicating that this regulation occurs post-translationally [95].

Beyond the COP1-centered regulatory module, the fifth subunit of the COP9 signalosome (CSN), SlCSN5-2, also contributes to the post-translational regulation of anthocyanin biosynthesis by modulating the stability of SlBBX20 [73]. Silencing *SlCSN5* leads to substantial anthocyanin accumulation in transgenic tomato calli and seedlings, whereas overexpression of SlCSN5–2 reduces anthocyanin levels. Mechanistically, SlCSN5-2 promotes the proteasomal degradation of SlBBX20 by enhancing its ubiquitination. Co-expression of SlCSN5-2 and SlBBX20 increases SlBBX20 ubiquitination and decreases its protein abundance [73]. Additionally, silencing the SlCSN5-2 ortholog *NbCSN5B* in tobacco significantly increases SlBBX20 protein accumulation, confirming the conserved role of CSN5 in regulating SlBBX20 stability. SlCSN5-2 likely exerts this function by modulating the activity of the CUL4–DET1–DDB1 Cullin–RING ligase (CRL) complex, which has previously been shown to ubiquitinate SlBBX20 [73]. Jasmonic acid (JA) and its derivative methyl jasmonate (MeJA) also regulate anthocyanin biosynthesis through the post-translational modulation of JAZ repressor proteins. JA signaling is perceived by the F-box protein CORONATINE INSENSITIVE1 (COI1), which recruits JAZ repressors to the SlCOI1 E3 ubiquitin ligase complex for polyubiquitination and proteasomal degradation [96–98]. In the absence of JA, JAZ proteins interact directly with various JA-responsive TFs, including components of the MBW complex, maintaining them in an inactive state and thereby inhibiting anthocyanin synthesis [99, 100]. Upon JA/MeJA treatment, JAZ proteins are degraded, releasing the MBW complex to activate the expression of anthocyanin biosynthetic genes and promote anthocyanin accumulation [38]. Interestingly, MeJA treatment does not significantly alter *SlJAF13* expression but increases SlJAF13 protein accumulation [38], suggesting that JA signaling may stabilize SlJAF13 at the post-translational level, although the precise mechanism warrants further investigation.

## Conclusion and prospective

**Figure 4:**
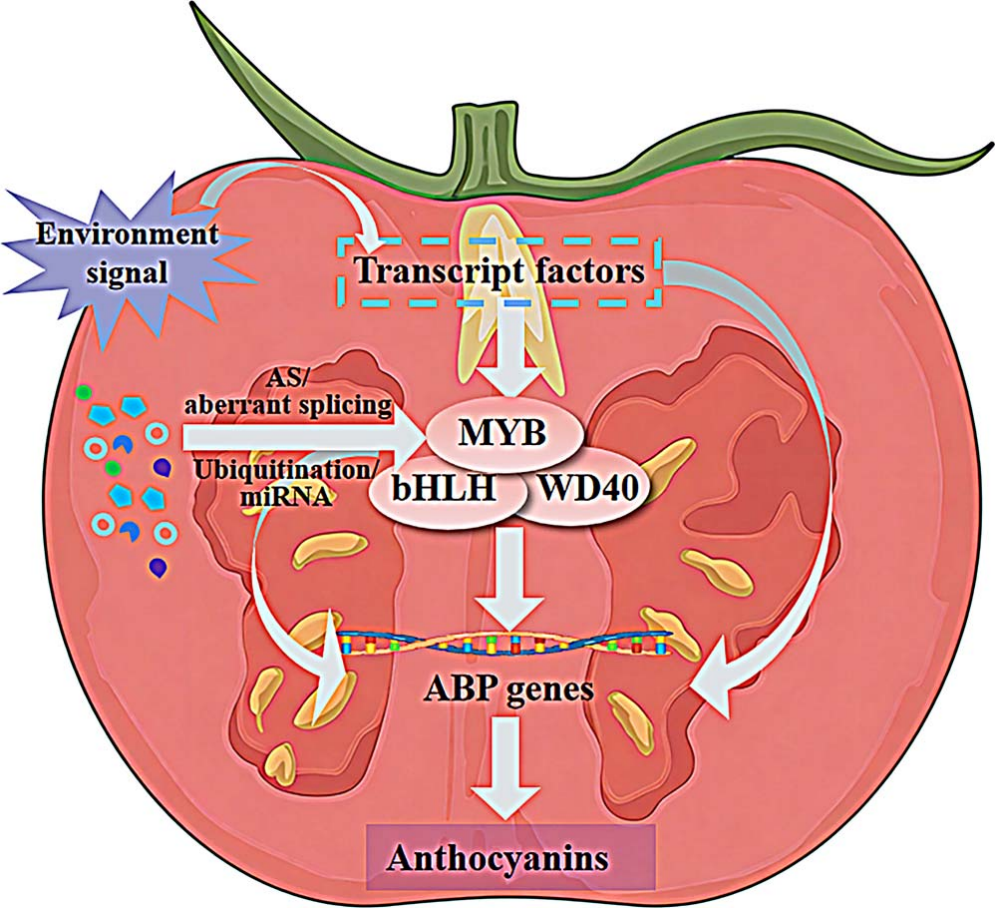
A regulatory model of anthocyanin biosynthesis in tomato. Environmental signals are perceived and transduced to activate specific TFs. These factors then induce the core regulatory hub, the MYB-bHLH-WD40 (MBW) transcription complex. In concert with the MBW complex, specific post-transcriptional and post-translational modifications modulate the expression of anthocyanin biosynthetic pathway (ABP) genes, ultimately driving anthocyanin synthesis and accumulation.

This review systematically summarizes the molecular regulatory mechanisms underlying anthocyanin biosynthesis in tomato. The process initiates from phenylalanine and proceeds through the phenylpropanoid pathway, where a series of EBGs and LBGs biosynthetic genes encode enzymes that catalyze the formation of stable anthocyanin compounds, which are subsequently transported and accumulated in vacuoles. Anthocyanin accumulation and composition are precisely controlled at multiple levels, including transcriptional regulation, post-transcriptional modification, and post-translational regulation. The MBW transcriptional activation complex (comprising MYB, bHLH, and WD40 proteins) plays a central role by activating the expression of downstream structural genes to promote anthocyanin synthesis. This process is further fine-tuned through alternative splicing, miRNA-mediated regulation, and ubiquitin-dependent protein degradation. Additionally, TFs such as SlHY5 and SlAREB1 (bZIP family), along with SlBBX20, SlBBX24, and SlCOL1 (BBX family), act as critical integrators. They relay diverse environmental signals including light, temperature, ABA, and low phosphate into the regulation of the anthocyanin biosynthetic pathway (Fig. 4). The in-depth dissection of these mechanisms provides a theoretical foundation for understanding fruit coloration and enhancing anthocyanin content. Anthocyanin accumulation results from the synergistic regulation of TFs, post-translational modifications, endogenous hormones, and environmental factors, highlighting the remarkable complexity of this metabolic pathway. Further elucidation of these regulatory mechanisms holds significant promise for breeding tomato varieties with high anthocyanin content, which would not only improve fruit appearance but also enhance nutritional value and stress adaptability.

Notably, significant gaps remain in our functional understanding of many anthocyanin-regulating TFs. For instance, the precise functions and mechanisms of action of genes such as *SlANT1-*like and *SlMYBTV-*like require definitive validation, ideally through the generation of loss-of-function mutants using CRISPR/Cas9-based gene editing. This technology offers substantial potential for precisely improving anthocyanin biosynthesis in tomato. Targeted editing of key TFs, structural genes, or regulatory miRNAs may allow directed enhancement of anthocyanin content and composition. However, it is important to note that increased anthocyanin accumulation may sometimes coincide with elevated organic acid levels, leading to undesirable fruit flavor and astringency. Therefore, tomato breeding must emphasize the balanced improvement of multiple quality traits. Hybridization of high-anthocyanin materials with cultivars possessing superior sugar-acid ratios and flavor profiles will be a crucial strategy for integrating desirable traits in future breeding programs. Moreover, evidence suggests that plants may have evolved mechanisms to coordinate different pigment synthesis pathways. Exploring whether similar regulatory networks exist in tomato and elucidating their molecular basis remain important research objectives.

Although significant progress has been made in understanding anthocyanin metabolism in tomato, several key scientific questions remain unanswered:

(1) Systematic elucidation of the crosstalk mechanisms in the anthocyanin regulatory network: The synergistic interactions between TFs and phytohormone signaling are not fully understood. For example, further mechanistic insight is needed to explain how ethylene induces *SlAN2-*like expression and whether it is involved in stress-induced expression of genes such as *SlAN2*.
(2) Elucidating the spatiotemporal dynamics of post-translational modifications of core regulators: The spatiotemporal dynamics and upstream signaling of post-transcriptional and post-translational modifications such as alternative splicing and ubiquitination on the core TFs in tomatoes still need to be further elucidated. Furthermore, post-translational modifications such as phosphorylation remain a significant knowledge gap.
(3) Engineering modification for anthocyanin compartment-specific accumulation: For precision breeding of anthocyanin-enriched tomatoes, achieving tissue and subcellular compartment-specific accumulation is an urgent need. This goal necessitates the identification of specific transporters and regulatory modules that control subcellular localization and fine-tune tissue-specific biosynthesis pathways.
(4) Elucidating the molecular basis of the trade-off between flavor and anthocyanin biosynthesis: It is important to note that anthocyanin metabolic engineering often leads to unintended alterations in tomato fruit flavor. However, the underlying genetic and metabolic crosstalk mechanisms driving this effect remain poorly understood.

In conclusion, while substantial advances have been made in elucidating the molecular basis of anthocyanin biosynthesis and regulation in tomato, this field still offers ample opportunities for further exploration. Addressing these questions will not only deepen our understanding of the regulation of plant secondary metabolism but also accelerate the development of new tomato varieties with improved nutritional value, enhanced visual appeal, and greater resilience to environmental stress.

## Data Availability

No data was used for the research described in the article.
